# Two cases of pyrocarbon capitate resurfacing after comminuted fracture of the capitate bone

**DOI:** 10.1080/23320885.2020.1834398

**Published:** 2020-12-24

**Authors:** Aleid C. J. Ruijs, Joël Rezzouk

**Affiliations:** a Department of Orthopaedic surgery, CHWAPI, Tournai, Belgium; b Centre Aquitain de la Main et du Poignet, Dax, France

**Keywords:** Capitate fracture, Fenton’s syndrome, pyrocarbon capitate prosthesis, RCPI

## Abstract

We present two cases of the use of a pyrocarbon capitate resurfacing implant (RCPI) after comminuted capitate fracture. Both cases were young males with a high-energy injury to the wrist. Follow-up was 21 and 29 months. Wrist ROM was decreased to about 47% and post-injury pain was limited.

## Introduction

Pyrocarbon Capitate Resurfacing has been primarily used for capitate resurfacing in cases of proximal row carpectomy for degenerative wrist diseases [1–3]. Our positive experience with the RCPI (resurfacing capitate pyrocarbon implant) prosthesis in elective cases has led us to consider its use also in comminuted capitate fractures. To share our experience, we would like to present these two cases.

## Case report

**Figure 1.**
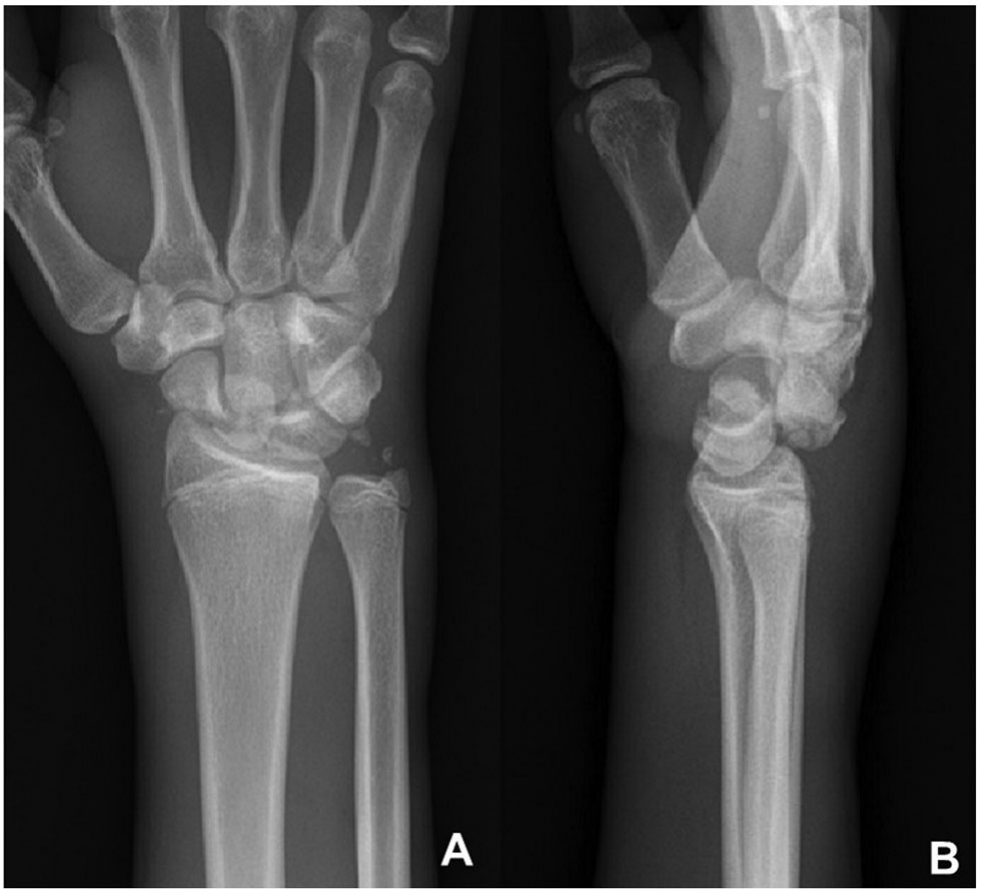
(A and B) Pre-operative x-rays of the first case.

**Figure 2.**
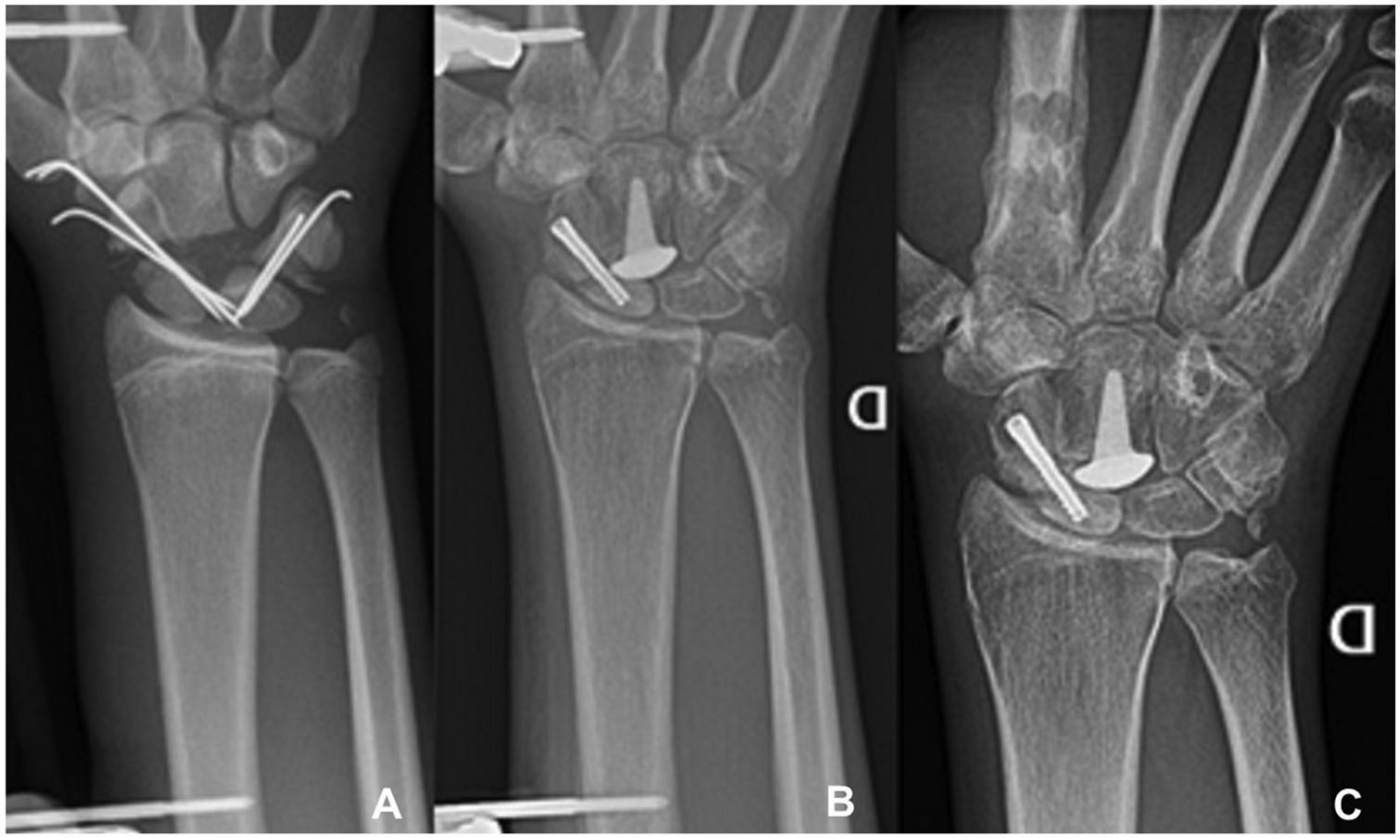
(A) X-ray after the first surgery. Note the empty space at the place of the proximal pole of the capitate. (B) X-ray after the second surgery, the K-wires were replaced by the RCPI prosthesis and a screw was inserted in the scaphoid. (C) At 21 months’ follow-up (all case 1).

The first case (NJ) is an 18-year-old male right-hand dominant student, who presented with a traumatic injury of his right wrist after a scooter accident. On admission, radiographies (Figure 1(A,B)) showed a perilunate dislocation with associated fracture of the scaphoid waist and proximal pole of the capitate, also known as scapho-capitate syndrome or Fenton’s Syndrome [4]. There was also an avulsion fracture of the ulnar styloid. On the day of injury, an open reduction and temporary fixation were carried out. Using a dorsal approach, we found a marked comminution of the proximal pole of the capitate, a complete tear of the lunotriquetral ligament, a grade IV cartilage lesion at the level of the dorsal side of the lunate facet of the distal radius and a scaphoid waist fracture. K-wires were used to stabilize the scaphoid fracture, the lunotriquetral ligament was primarily sutured, and a spanning external fixator (PoingFIX wrist fixator, Small Bone Innovations, Morrisville, PA, US) from the radius to the metacarpal level was placed to provide temporary distraction at the carpal level. The remnants of the proximal pole of the capitate were removed (Figure 2(A)).

Six weeks later, the patient underwent a second intervention. The second surgery would ideally have been performed earlier, but the patient was not available for surgery at that time. The scaphoid fracture was treated with a corticocancellous bone graft from the iliac crest and a compression screw (Orris, Avignon, OSD, France). The head of the capitate was replaced using a RCPI pyrocarbon prosthesis (Tornier, Saint-Ismier, France) [1,2]. Distraction was maintained for another three months, in order to protect the scaphoid from the pressure of the RCPI prosthesis and thus allowing stable incorporation of the bone graft (Figure 2(B)). After removal of the external fixator, the patient started hand therapy for four to five times a week.

At 21 months’ follow-up (Figure 2(C)), the patient had a painless wrist with good motion. He had returned to his studies and resumed sports activities. On examination, his ROM was 45 degrees of flexion and 15 degrees of extension of the wrist, and symmetrical pronation and supination of both 85 degrees. Ulnar and radial deviation were nearly normal at respectively 40 and 10 degrees compared to respectively 60 and 20 degrees of the left wrist. Compared to the left hand, squeeze strength was 40% (Saehan Squeeze dynamometer, Saehan Medic, Republic of Korea), Jamar grip strength 49% and key pinch strength 73%. He was not experiencing any pain, with a Quick DASH score of 25% (possible score between 0% and 100%, a higher score indicates a greater disability), and a score of 6/100 on the Patient Rated Wrist Evaluation (PRWE). This scale measures wrist-pain and disability in activities of daily living, the best score is zero, the worst score is 100.

**Figure 3.**
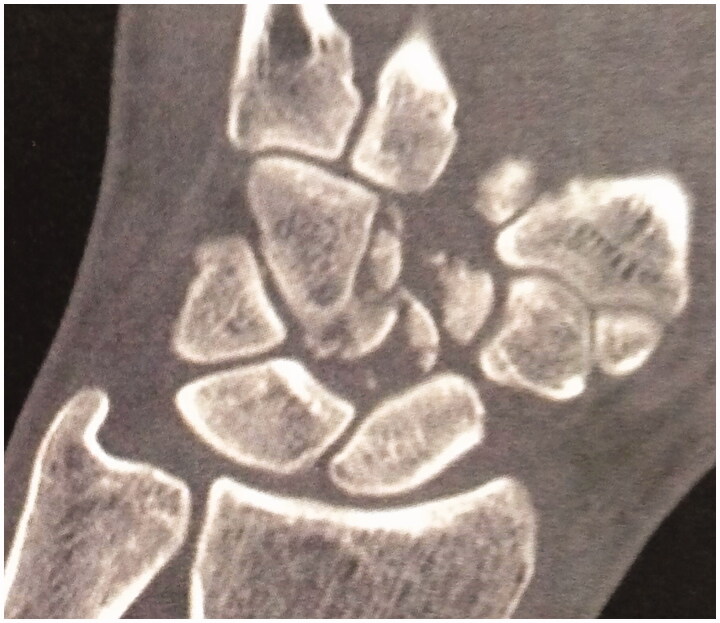
Pre-operative CT-scan case 2.

**Figure 4.**
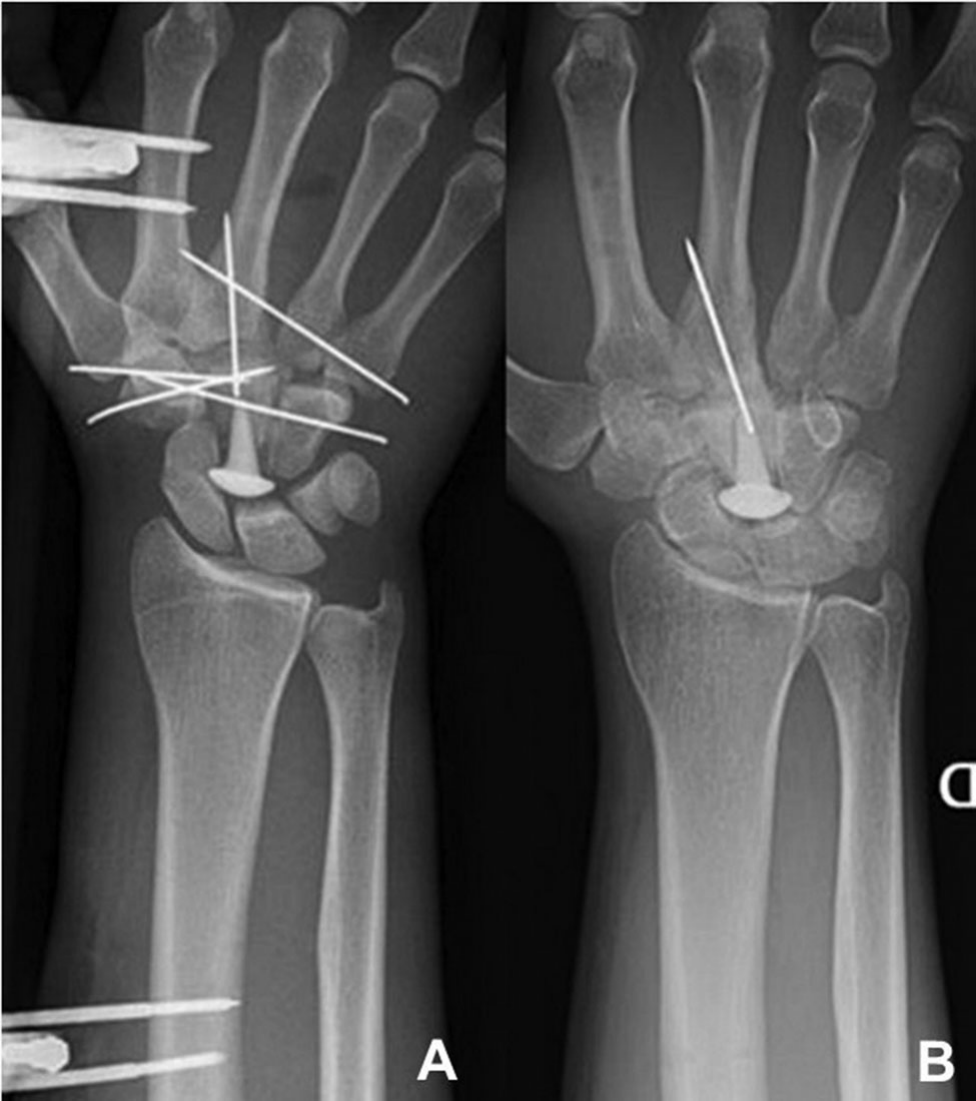
(A) Post-operative x-ray case 2. (B) X-ray at 29 months’ follow-up, after removal of the external fixation and K-wires.

The second case (LG) is that of a 20-year-old male right-hand dominant student, who presented after a skiing accident with a traumatic injury of his right wrist. Standard X-rays and a CT scan showed a comminuted fracture of the capitate (Figure 3). During surgery, we noted that the capitate was severely comminuted, and there was extensive cartilage damage between the capitate and hamate, and capitate and trapezoid. The comminuted pieces of the capitate were removed and replaced by a corticocancellous bone graft harvested from the iliac crest. The volume needed was measured before harvesting the bone graft. It was a corticocancellous bone graft in one piece. The bone graft was prepared with a 75 degrees angle on the dorsal side, which corresponds to the 15-degree angle of the distal radius. A central tract was made in the graft with a large K-wire. Then the broach was inserted into the bone graft. The implant was placed into the graft, and the graft with implant was then fixed by several K-wires to the other carpal and metacarpal bones. Cancellous bone was added between the trapezoid bone, the bone graft and the hamate bone. K-wires were added for stability. There was good contact and mobility between the RCPI prosthesis and the scaphoid and lunate bones. A spanning external fixator was applied to provide distraction for protection of the cartilage during bone healing (Figure 4(A)). It was removed at 6 weeks post-operatively. At 29 months’ follow-up, his ROM was 55 degrees of flexion and 45 degrees of extension of the wrist, and a loss of both pronation and supination of 15°. Ulnar and radial deviation was functional at respectively 45 and 10 degrees compared to 50 and 30 degrees of the left wrist. Squeeze strength was 100%, Jamar grip strength was 65% and key pinch strength was 91% compared to the left side. At rest, he had no pain in his wrist, and after activity, his maximum pain level was 4 out of 10 (VAS-scale). The Quick Dash showed a score of 6%, and the PRWE a score of 13.5 out of 100. The RCPI prosthesis was in place and there were no signs of secondary degenerative changes between the prosthesis and the scaphoid or lunate bones (Figure 4(B)). He has had to give up his climbing activities but does still ride his motorcycle.

## Discussion

Perilunate dislocation with fracture of the scaphoid and capitate is a rare but serious injury, leading in general to an important loss of function and early degenerative changes. In most cases, the treatment is emergency open reduction and fixation, but if there is a bony defect of the head of the capitate, a four-corner arthrodesis is performed. In four-corner fusion one can expect a grip strength and flexion and extension of 50%, comparable to our two cases, and 40% of radioulnar deviation where we had near normal results [5]. Pyrocarbon is a ceramic material, which has been used in the medical field since the 1960s. It was first used as prosthetic heart valves, then MCP and PIP joint implants and most recently wrist bone implants [6]. The July 2014 (J Hand Surg Eur. 2014; 39) issue of The Journal of Hand Surgery (European Volume) contains six full length articles, one editorial comment, one commentary, and one short report, all relevant to pyrocarbon and pyrolytic implant and prosthesis usage in the hand. The pyrocarbon RCPI prosthesis has been used for chronic degeneration of the wrist as in SNAC or SLAC wrist, in combination with a proximal row carpectomy [1,2]. In these cases, a four-corner fusion or capitolunate arthrodesis was not possible because of osteoarthritis of the radiocarpal joint. Another recent article compares the results of a proximal row carpectomy with RCPI implant (PRC-RCPI) and a four-corner arthrodesis (4CA) for advanced carpal arthritis [3]. The first group had 31 patients and the second group 26. Results were comparable between both groups. The RCPI prosthesis has also been used for resurfacing of the capitate head without adjacent proximal row carpectomy. Dereudre et al. [7], present a case in which an avascular necrosis of the capitate in a 30-year-old woman was successfully treated with a RCPI prosthesis. Although experiencing persistent decrease in range of motion, she had resumed her normal activities at 22 months’ follow-up. Another recent article describes two cases in which a chronic Fenton’s syndrome [8] was treated with the same prosthesis, giving a good outcome at 48 and 64 months’ follow-up respectively. A longer follow-up and more cases are needed to better assess the use of this prosthesis in comminuted fractures of the capitate. However, in this preliminary case report with a relatively short follow-up, the results are satisfactory and without complications. There were no complications due to the use of the external fixator or at the site of the bone graft (iliac crest). The use of a pyrocarbon capitate resurfacing in comminuted fractures of the capitate may be considered as a treatment alternative to a four-corner fusion [9]. In the case of failure, a secondary arthrodesis can still be performed.
